# A ‘He Awa Whiria’ approach: integrating Māori knowledge and cultural values into audiological research and hearing health services

**DOI:** 10.1080/03036758.2024.2381753

**Published:** 2024-08-19

**Authors:** James Dawson, Jennifer Smith, Greg A. O’Beirne, Alehandrea Raiha Manuel

**Affiliations:** a School of Psychology Speech and Hearing, Te Whare Wānanga o Waitaha, The University of Canterbury, Ōtautahi, New Zealand; b Child Well-being Research Institute, Te Whare Wānanga o Waitaha, The University of Canterbury, Ōtautahi, New Zealand; c School of Population Health, Faculty of Medical and Health Sciences, Waipapa Taumata Rau, The University of Auckland, Tāmaki Makaurau, New Zealand; d Eisdell Moore Centre, Waipapa Taumata Rau | The University of Auckland, Tāmaki Makaurau, New Zealand

**Keywords:** Audiological health, indigenous, language revitalisation, culturally appropriate health care, hearing testing

## Abstract

Indigenous peoples of Aotearoa New Zealand, Māori, are often excluded and underserved by hearing health services that often neglect their cultural values and needs. In response to this, we aimed to advance the delivery of culturally appropriate services with Māori clients through the validation of a te reo Māori hearing screening test. A He Awa Whiria: Braided rivers framework was applied to weave Māori knowledge and cultural values into this audiological research and to support the collaboration of Māori and non-Māori researchers through a Kaupapa Māori approach stream. While the validation of the screening test was interrupted due to COVID-19, we discuss in detail the approach stream used in this study. Together the approach and validation streams form the foundation of an ongoing research initiative aimed at addressing Western biomedical approach limitations, expanding the hearing health sector's knowledge base to deliver culturally responsive care, and in a small way contributing to the revitalisation of te reo Māori.

**Glossary of Māori words:** Ahitereiria ki te Hauāuru: Western Australia; atua: ancestor with continuing influence; Aotearoa: North Island of New Zealand - now used as the Māori word for New Zealand; hapū: sub-tribal, clans, wider whānau descent or political units; hongi: traditional Māori greeting; He Awa Whiria: braided rivers approach; hui: meeting; ia: Te reo Māori pronoun for everyone; iwi: tribe; kai: food; Kai Tahu/Ngai Tahu: tribal group of the South Island; kanohi kitea: the seen face; kanohi ki te kanohi: face-to-face; karakia: incantation, chant, thought, or prayer; kaumātua: Māori elder/s; kaupapa: topic, policy, matter of discussion; Kaupapa Māori: a philosophical approach using Māori knowledge and values; kete: woven basket/s; Kohanga Reo: Māori language preschool; kōrero: narratives and prose; koringo-tangi-roa: the wooden flute or trumpet of Ngongo; kūmara: wweet potato; Kura Kaupapa: Māori language immersion schools; kupu: word, vocabulary; manaakitanga: the process of showing respect, generosity and care for others; Māori: Indigenous people of Aotearoa New Zealand; marae: a significant Māori community complex of buildings; mātauranga Māori: Indigenous Māori knowledge; Ngā Rauru: an iwi of Taranaki; Ngāpuhi: a tribal group situated in the top of the North Island; Ngāti Hau: people of the Whanganui River; Ngāti Porou: a tribal group of the East Coast of the North Island; Ngāti Whātua: tribal group between the Hokianga Harbour and Tāmaki; Ōtautahi: traditional name known now as Christchurch; Pākehā: New Zealander with European descent, non-Māori; Papatūānuku: Earth mother; pihoi: inattentively deaf; pūwharawhara: deaf; Rakinui/Ranginui: Sky father; Rehua: a deaf atua associated with kindness, a child of Pāpātuanuku and Rakinui; tāhorehore: deaf as if the ears were cropped; Takaroa/Tangaroa: an atua of the sea; Taki-ao-terangi: daughter of Rehua; Tāmaki: Auckland; Tāne-tūturi: a deaf child of Ranginui and Papatūānuku; Tangata Tiriti: people of Te Tiriti, referring to all non-Māori citizens and residents of Aotearoa; tangata whenua: people of the land, Indigenous peoples, local people, hosts; taonga: treasure of social or cultural value; tapu: sacred, spiritual restriction containing a strong imposition of rules and prohibitions; taringa kōhatu: deaf, stubborn; taringa muhu kai: ear listening for food - a person whose only concern is when food is served; taringa puta iti: little ear orifice, inattentive; taringa turi: deaf; teina: young, less skilled learner; te Ao Māori: the Māori world; te reo Māori (te reo): the Māori language, Indigenous language of Aotearoa; Te Tiriti o Waitangi: Signed in 1840 this is the Māori language version of an agreement between two internationally recognised sovereign nations, Māori, as tāngata whenua, and the British Crown; Te Waipounamu: the South Island of Aotearoa New Zealand; Te Whakamātautau Whakarongo o Aotearoa: Te Reo Māori Digit Triplet Test, TRMDTT; tikanga Māori: Māori customs, procedures, protocols, practices, system of values, the Māori way; tīpuna: ancestors; tohunga: priest, practitioner, expert; tuakana: mentors; turi: deaf; turikere: deafened; waiata: songs and chants; wairuatanga: spiritual well-being; Waitaha: traditional name for region known as Canterbury; wānanga: to meet and discuss, Māori learning, educational forum; whakamā: shame, embarrassment, guilt; whakapapa: To place in layers, to recite genealogies, lineage; whakarongo: an intransitive verb that can mean to listen but may also be used to ready the senses such as to smell, touch or feel; whakataukī: proverb, significant saying; whakawhanaungatanga: an indigenous process of creating relational connection; whānau: immediate and extended family and friends.

## Introduction

In Aotearoa me Te Waipounamu New Zealand (Aotearoa) Māori are frequently underserved by ‘one-size-fits-all’ health services rooted in the dominant culture's values. Because of this ideology, there are significant disparities in outcomes for Māori relative to non-Māori populations nationally across almost all health and social indicators. In audiology, hearing health workers often overlook Māori cultural values, leaving many Māori feeling alienated, whakamā (ashamed and embarrassed), and unsafe (Lowe 2023). Consequently, there are significant inequities in hearing health outcomes for Māori, and greater unmet needs relative to non-Māori.

Improving audiological service delivery in Aotearoa to ensure widespread provision of culturally safe hearing healthcare for Māori is imperative, and forms the focus of this paper and the respective companion papers (Lowe et al. 2024; Manuel et al. 2024). The development of audiological assessments in te reo Māori (te reo) is a vital component of this service provision. This paper has its origins in the Master of Audiology thesis of Dawson (2022), which aimed to continue the development of a digit triplet test (DTT) for hearing screening in te reo known as ‘Te Whakamātautau Whakarongo o Aotearoa’ or ‘Te Reo Māori Digit Triplet Test’ (TRMDTT).

The cultural importance of te reo cannot be overstated. Te reo is frequently referred to as the ‘cornerstone’ of what it means to be Māori and is a taonga (a treasure of social or cultural value). Te reo is a medium through which Māori express their worldview and an opportunity for non-Māori to understand Māori culture and perspectives better. Ngāpuhi leader and co-patron of the Kōhanga Reo (Māori language preschool) movement, Sir James Hēnare, once stated, “Ko te reo te kaipupuri i te Māoritanga – the Māori language is the repository of Māori culture’ (cited in Mikaere and Hutchings 2013, p. 13).

Settler colonialism and numerous harmful acts of legislation and government policies led to a significant decline in the use of te reo and an increase in the use of English. In 1972, a petition to reintroduce te reo into schools was the catalyst to numerous Māori-led revitalisation efforts that successfully reversed the decline in the language (Hardman 2018). Over time and in partnership between government and communities, te reo revitalisation has become more coordinated and strategic, featuring formal language policies and planning (Hardman 2018; De Bres, 2008).

It is argued that ‘legitimation and institutionalisation’ are required for te reo to become ‘a language of the public realm’ (Ahu, 2012 as cited in Hardman 2018, p. 46). Thus, it is timely for hearing healthcare to join this movement in part through the provision of te reo assessments, such as TRMDTT. Increasing the availability of hearing screening resources and assessments in te reo acknowledges its status as an official language of Aotearoa, and contributes to normalising the language in a healthcare context as part of broader language revitalisation efforts.

The development of the TRMDTT has been an ongoing and incremental process, with several researchers making essential contributions before this study. TRMDTT was initially developed in 2011 (Murray 2012; Bowden 2013), and was the first DTT to be created in a non-European indigenous language. As with other DTTs (Smits and Houtgast 2005), it uses the digits 0–9 presented in background noise to reliably screen hearing. However, unlike its New Zealand English counterpart (the NZDTT; King 2011), the development of the TRMDTT had stalled in the verification stage as the research team had difficulty recruiting hard-of-hearing te reo speakers required to allow robust pass-refer criteria to be determined. Completing the validation of the TRMDTT increasingly became a priority, but it was clear that further research needed to incorporate a Kaupapa Māori, ‘by Māori, for Māori, with Māori’ approach (Pipi et al. 2004)

A He Awa Whiria: Braided rivers framework (Macfarlane and Macfarlane 2019) was employed to support both Māori and non-Māori researchers to collaborate in innovative ways. The review of literature presented in this paper was conducted to understand the complex foundation on which the project sat. From the resulting team wānanga (To meet and discuss, Māori learning, educational forum), the project was structured into two interwoven streams. First, an approach stream – a Kaupapa Māori approach exploring how Māori knowledge and cultural values could be integrated into audiological research and mainstream audiology services. Second, the validation Stream – a pilot study to validate and improve the performance of the TRMDTT.

The validation stream ended prematurely due to COVID restrictions but has since been completed by a University of Canterbury MAud student Tanya Neame (Te Atiawa). The results of this validation will be published separately, and are beyond the scope of this paper, which instead focuses on the approach stream in the hope that it may serve as a guide for future research developing Māori hearing assessments.

## Background on Māori hearing loss

To better understand the background context of Māori hearing loss and hearing health in Aotearoa, we sought to consider how common hearing loss and deafness were in traditional Māori society, and how hearing loss and deafness were perceived by whānau (extended family and friends), hapū (sub-tribal, clans, wider whānau descent or political units), and iwi (tribe). We summarise here the relatively scarce historical accounts of hearing loss pre-colonisation, traditional perspectives of hearing loss and deafness, and describe the current reality of hearing health for Māori.

### Written accounts of hearing loss

Passing observations and eyewitness accounts of primary European sources relating to Māori health and well-being are spread widely throughout a variety of documents such as journals, ship's logs, diaries, and letters, written by visitors to Aotearoa, but are generally unreliable, having been written by people who lacked knowledge of Māori culture or had no prior medical training. Moreover, available literature on Māori health between 1840 and the 1900s is subject to European cultural bias, having been authored by non-Māori historians, anthropologists, missionaries, and ethnographers. We therefore viewed this literature through a critical lens.

#### Ancestral stories

Māori culture has a strongly developed tradition of intergenerational transfer of knowledge through oral language via whakapapa (to place in layers, to recite genealogies, lineage), whakataukī (proverb, significant saying), kōrero (narratives and prose) and waiata (songs and chants) (McRae 2017). The following two oral traditions feature hearing loss.

The first relates to the origin of hearing loss and deafness from Kai Tahu (also known as Ngai Tahu). In Tiramōrehu et al. (1987), the tohunga (priest, practitioner, expert) Matiaha Tiramōrehu from Moeraki retold the Kai Tahu creation story involving the deities Rakinui (sky father), Papatūānuku (earth mother) and Takaroa (an atua [ancestor with continuing influence] of the sea). According to Southern traditions, the story begins with a partnership between Papatūānuku and Takaroa. Takaroa leaves to bury their child's placenta. Takaroa returns to discover that in his absence, he has lost his wife Papatūānuku to Rakinui, and this partnership has produced several children. After battling and wounding Rakinui, Takaroa cursed these offspring and the children were born with congenital anomalies and sensory losses. The parents named each of the children according to their condition, including a deaf child called Tāne-tūturi.

The second oral tradition, from the North Island iwi of Taranaki, describes one of the first treatments for hearing loss and deafness. In 1887, the English ethnographer John White (White 2001) recorded two versions of a story originating from North Island iwi Ngāti Hau and Ngā Rauru, involving the deaf atua Rehua, the eldest child of Papatūānuku and Rakinui. Rehua is a powerful atua associated with kindness. According to these stories, the ears of Rehua either were or became deaf. Another party named Ngongo (or Ngo) blew into Rehua's ears with his wooden flute or trumpet named Koringo-tangi-roa. After a considerable time had passed, Rehua's deafness was ‘cured’. Rehua was so delighted that he could hear the voice of man and all of the world's sounds, that he gifted his daughter Taki-ao-te-rangi to Ngongo as a wife.

### Kupu Māori

Māori have several kupu Māori (Māori word/s) and idioms relating to the ears, hearing loss, and deafness, many with subtle differences in meaning and expression (The Review Team 1989; Forman 2003). These include Turi (Deaf); taringa turi and pūwharawhara (deaf); Turikere (deafened); Pihoi (inattentively deaf); Taringa kōhatu (deaf, stubborn); Tāhorehore (deaf as if the ears were cropped); Taringa puta iti (little ear orifice, inattentive); and Taringa muhu kai (ear listening for food – a person whose only concern is when food is served).

Context is essential for understanding the meaning of kupu Māori – for example, ‘whakarongo’ is an intransitive verb that can mean to listen but may also be used to ready the senses such as to smell, touch or feel. From the various terms used, it is apparent that Māori recognised different degrees of hearing loss and deafness and had developed beliefs regarding its probable cause(s).

#### Traditional attitudes

Forman (2003) acknowledges the paucity of written evidence or known oral traditions and suggests that it is likely that Māori with hearing loss were generally tolerated and accepted in traditional pre-Tiriti (treaty) society. Forman speculated that providing that the body was sound, deaf and hard of hearing, individuals could still participate in essential activities such as hunting, fishing, child-minding, arts and crafts, and warfare (as required). By contrast, Tikao et al. (2009) found that in the ancient world, Māori with sensory losses were considered to be more closely connected to atua. Tikao et al. (2009) contend that there was a greater appreciation of their talents and strengths rather than their deficits. Sensory losses and other physical differences were regarded as indicators of greatness.

According to Hickey and Wilson (2017), the concept of disability did not exist for Māori before European colonisation. It is likely that with the advent of colonisation and the imposition of Western medical models, traditional attitudes of Māori towards people with hearing loss have become overshadowed by Western constructs of hearing loss as a disease or disability (Smiler and McKee 2006). This dominant paradigm of hearing loss as a physical disability has continued for many years until recent shifts towards social constructs that acknowledge societal stigma (Oliver 1996).

#### The current reality of hearing health for Māori

The impacts of unaddressed hearing loss are felt across many aspects of modern life: communication, cognition, education, employment, isolation, mental health, and relationships. Despite the significant impact, Bird and O'Beirne (2015) observe that in Aotearoa, hearing loss is ‘under-reported, under-diagnosed and generally underrated by society’ (pg. 6). Additionally, Māori-specific hearing data is scarce – a long-standing and unaddressed national issue highlighted three decades ago in the 1989 ‘Whakarongo Mai’ report (The Review Team 1989).

#### Prevalence and economic costs of hearing loss

The 2013 Disability Survey suggested a national prevalence of hearing loss at 8% for Māori and 9% for non-Māori (Statistics New Zealand 2015). However, after analysing the 1991–1992 Census data and controlling for the reduced life expectancy of Māori, Greville (2005) suggested a higher prevalence for Māori at 12.1%. Further, a Deloitte Access Economics (2017) report for the National Foundation for the Deaf estimated a national prevalence of hearing loss of 18.9%, contending that the self-reported data collected via the Disability Survey was likely to be an underestimate.

The same report estimated that national hearing loss-related expenditure (including hospital, health professional, out-of-hospital medical, pharmaceutical, implant, and research costs) was $131.8 million for all New Zealanders in 2016 (Deloitte Access Economics 2017), of which approximately 10% were paid directly by individuals and their whānau as out-of-pocket expenses. However, these economic costs are significantly lower than the estimated costs due to loss of well-being from hearing loss; for 2016, this was estimated at 23,130 disability-adjusted life years or $3.9 billion (Deloitte Access Economics 2017). A New Zealand Institute of Economic Research (NZIER) report commissioned by the New Zealand Hearing Industry Association estimated that mitigating the effects of hearing loss at work would lead to annual increases in real GDP in the billions through increased labour productivity and increased employment while mitigating the social impacts of hearing loss in the elderly would also contribute hundreds of millions of dollars to the economy annually (NZIER 2023).

#### Identification of hearing loss across the life course

The benefits of early identification of hearing loss and timely interventions among children are well known, leading to a high rate of newborn hearing screening in higher-income countries (Neumann et al. 2022). However, adult hearing health screening is often overlooked. Except for mandatory workplace screening for certain occupations or individuals taking the initiative, no routine hearing screening assessments are offered to the adult population in Aotearoa. Consequently, hearing loss associated with aging, illness, injury, or genetic factors can remain undetected and unassisted for years.

Kaumātua (Māori elder/s) are well recognised as the repositories of Mātauranga Māori (Indigenous Māori knowledge) to be passed down through generations. There is however a higher prevalence of hearing loss among kaumātua compared to the total New Zealand population aged over 65 (32% c.f. 28%) (Statistics New Zealand 2014). Exeter et al. (2015) estimate the prevalence of hearing loss for all New Zealanders aged over 70 years will triple between 2011 and 2031. The increased prevalence of hearing loss in this cohort will likely inhibit the participation of kaumātua to fulfil specific oratory roles and obligations. Overseas studies have shown earlier identification of hearing loss through screening results in hearing aids being fitted to younger people and those with less than severe hearing loss, giving up to 10 additional years of benefit relative to those individuals who initiate hearing aid use later in life (Davis et al. 2007).

## Methodology

He Awa Whiria (Braided rivers approach) informs the methodology used for the present study and weaves together two very different knowledge systems – Mātauranga Māori and Western knowledge systems.

### Braiding bodies of knowledge

Mātauranga Māori is an umbrella term used to describe a vast and complex body of indigenous knowledge (Mead 2012). Mātauranga Māori represents the totality of Māori knowledge and experience derived from generations of Māori tīpuna (ancestors) in Aotearoa and their ancestral homelands (Mercier et al. 2012). According to Mead (2016), Mātauranga Māori is a ‘super subject’ that encompasses ‘all branches of Māori knowledge, past, present and still developing’ (p.305). The responsibility of having said knowledge or knowing Mātauranga Māori is linked to collective well-being, the continuing existence of Māori as self-determining and sovereign people, and the rights to develop and represent their thoughts and imaginations from their own experiences (Smith et al. 2016).

Like other knowledge systems, Western knowledge is concerned with understanding the world we live in; it is the result of humans’ individual and collectively learned experiences. This form of knowledge is generally based on widely accepted and explanatory scientific theories developed using the scientific method. Western science informs the biomedical model and most medical research and healthcare activities in the Western world (Engel 1977). As Belanger (2018) observes, ‘[Western philosophy] continues to dominate university classrooms by offering what is considered to be the exclusive window for examining how we as people know what we know’ (p.3). Medical models typically conceptualise health as the state of being free from disease and are characterised by reductionist and individualist principles (Clark 2014). The discipline of audiology is no exception; despite the increasing use of the WHO’s ICF framework (a biopsychosocial model) in the training of audiologists, the biomedical model still informs a disproportionate amount of the language audiologists use to talk about their services, and the logic and methods used to diagnose and treat hearing loss (Duchan 2004; Meyer et al. 2016).

Numerous authors have compared Mātauranga Māori and Western Science. Durie (2004) observes that historically, the global dominance of Western Science and intolerance of Mātauranga Māori has seen the two knowledge systems pitted against each other to the detriment of both. Clarke (2006) asserts that the two knowledge systems are incomparable due to fundamental differences. In contrast, authors such as Stewart (2007) identify or seek common ground. Other works include convergence models by Mercier et al., and cultural interface approaches including that of Durie (2004), who argues that creativity, innovation, and new insights can be achieved by exploring the interface between the two systems and combining bodies of knowledge.

While the medical model has undeniable utility, there is scope for the audiology profession to challenge the dominance of Western scientific and biomedical approaches and to broaden its thinking regarding the delivery of culturally appropriate services for Māori clients. Western Science and Mātauranga Māori are not mutually exclusive; in the remainder of this paper, we give an example of how He Awa Whiria (Macfarlane et al. 2015) could be applied to audiological research to combine the best of both knowledge systems.

### He Awa Whiria – A braided rivers framework

The He Awa Whiria framework was created by Professor Angus Hikairo Macfarlane, Professor of Māori Research at the University of Canterbury. It features the metaphor of braided rivers, an iconic feature of Te Waipounamu and the Waitaha (Canterbury region) region, representing Mātauranga Māori and Western knowledge streams running alongside each other with equal strength. These rivers are characterised by numerous, ever-changing channels, with varying flows and shingle layers as they move over the landscape. The framework emphasises the point at which the two rivers converge (braid) and represents a space where collaboration and shared learning can occur, rather than assimilating one into the other. According to Macfarlane et al. (2019), although fundamentally sound, Western knowledge and theory are culturally bound and cannot be transferred directly into another (Indigenous Māori) culture. Macfarlane and Macfarlane (2019) assert that ‘it is inappropriate to seek solutions to indigenous challenges solely from within Western knowledge streams … [this framework] creates an approach that is potentially more powerful than either knowledge stream is able to produce unilaterally’ (p.12).

A ‘He Awa Whiria’ approach was particularly appropriate for this study which combines the Western science of audiology with Mātauranga Māori by a team of both European and Māori researchers. The Braided Rivers framework provided the study with inspiration and a roadmap for navigating the complexities of this cross-cultural project and projects moving forward.

**Figure 1.**
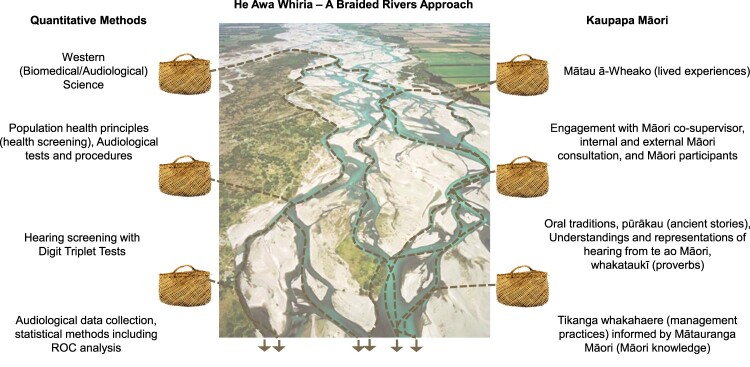
The He Awa Whiria framework adapted from Macfarlane et al. (2015). The kete (woven baskets) represent baskets of knowledge. The dashed lines represent a convergence of Indigenous Māori and Western science knowledge streams where shared learning occurs. Figure taken from Dawson (2022) Integrating Māori Knowledge and Cultural Values into Audiological Research and Hearing Health Services: An Approach Inspired by He Awa Whiria – A Braided Rivers Framework (Image sources: The Rakaia River – Homer (2015). Kete – Tauranga Heritage Collection 0224/85).

In Figure 1, the central image depicts the Rakaia River, typical of Canterbury's braided rivers. The kete (woven basket/s) on both sides of the river represent knowledge repositories. The approach stream flows from the right. The validation stream or quantitative methods stream flows from the left side of the diagram. Finally, the dashed lines emerging from the kete represent a convergence of Māori and Western knowledge where shared learning occurs.

#### The approach stream

The approach stream was enacted by a focus on core Māori cultural values (as described below), adherence to tikanga Māori (‘the Māori way’; Mead 2016), and the valuing of lived experiences of our research participants. The intent was to carry out this research using as close to a Kaupapa Māori approach as was possible for a non-Māori researcher. To this end, considerable time and effort were spent in discussions with tuakana (mentors) on how these principles, values, and protocols could be integrated into the clinical encounters throughout the data collection period, with non-Māori members of the research team working as allies and collaborators while ensuring the research remained under Māori control.

In this stream, we incorporated the ‘Community-Up’ approach developed by Smith (1999; 2021) and subsequent work on Kaupapa Māori research ethics by Cram (2001; 2009) and Smith (2006). As a result, the following kete of practices emerged (see table one), the first seven (Aroha ki te tangata; Kanohi kitea; Titiro, Whakarongo … kōrero; Manaaki ki te tangata; Kia tūpato; Kaua e takahia te mana o te tangata; Kia māhaki; Kia kaha Te Reo Māori) are directly from Smith’s Community-Up research practices. To these, the value of ‘Kia Kaha Te Reo Māori’ has been added (see Table 1).

**Table 1.** Application of the ‘Community-Up’ (CU) approach to audiological research.

*1. Aroha ki te tangata (respect for people)* was shown through Kotahitanga (unity, solidarity), Manaakitanga (Respect, generosity, hospitality – described further below), and Whanaungatanga/Whakawhanaungatanga (connectedness and the building of relationships). Kotahitanga was exemplified by Māori and non-Māori researchers, Māori research participants, and mentors working together towards a shared kaupapa (purpose, plan, initiative). Whanaungatanga/ Whakawhanaungatanga not only formed part of each session with research participants (with time taken for introductions and conversation before commencing clinical tasks), but from the outset was essential to build trust with the local community and facilitate recruitment. The lead author attended community-based TRM wānanga (Māori language classes) until the national COVID-19 lockdown was imposed, which provided the opportunity to meet TRM speakers, establish relationships, and build trust before asking individuals to participate. This kūmara vine led to invitations to present the study to kaumātua at local marae, community health hui and social events, Kura Kaupapa, District Health Boards, primary health services, and Māori and Pasifika health services, and enabled exchanging contact details with interested parties, and a way to share recruitment posters and participant information sheets. This approach led to introductions to several interested individuals, many of whom (including Dame Aroha Reriti-Crofts) helped enormously as champions for the study. They recruited their whānau and provided excellent advice and suggestions.
*2. Kanohi kitea* and kanohi ki te kanohi are similar Māori concepts concerning the act of being physically present and being seen to strengthen relationships and to convey a sense of commitment to a kaupapa (O'Carroll 2013). This in-person consultation allows the community to use all their senses for assessing and evaluating the advantages and disadvantages of participating (Cram and Pipi 2000). The research design for the present study accounted for this preference, where a face-to-face method was predominantly used to build relationships and recruit participants. The lead author met face-to-face with Māori organisations, groups, and individuals, giving presentations about the study, demonstrated the hearing screening tool, and answering questions as they arose. This increased trust by demonstrating accountability and commitment to the initiative. Images of the research team were included in all participant documentation for the study.
*3. Titiro, Whakarongo … kōrero (Look, listen … and maybe speak)* is the principle of developing an understanding before opening your mouth to speak, a process which helps researchers show respect and a willingness to learn (Pere 1982; Pipi et al. 2004). This meant when visiting marae and other spaces that it was important to assume the positionality of an outsider; following tikanga, being comfortable in being guided through as teina.
*4. Manaaki ki te tangata (Share and host people, be generous)*. Manaakitanga involved ensuring that a welcoming, hospitable environment was provided at all stages. During the recruitment phase, individuals and organisations were unconditionally gifted various forms of koha, including kai for meetings kanohi ki te kanohi with friends and new contacts, or cash contributions towards the cost of hosting. We invited questions before sessions, offering flexibility with appointments and testing environments (university clinic or community testing), encouraging participants to bring whānau members and support persons, offering travel assistance, fuel reimbursement vouchers, and free car parking. Participants and their whānau were greeted in te reo Māori and using the person's name (checking correct pronunciation), with a handshake, hongi, or COVID-suitable alternative. Kai, drinks, and rest breaks provided as needed during the session.
*5. Kia tūpato (Be cautious)*. Great care was taken to ensure a culturally safe environment, as well as appropriate safeguarding (as opposed to ownership) of all Māori data collected during the study. This guardianship role included appropriate, safe, and secure collection, storage, handling, and communication of all data. Participants were informed of their right to withdraw their consent to use any data collected.
*6. Kaua e takahia te mana o te tangata (Do not trample over the mana or dignity of people)*. As we were undertaking this kaupapa in Ōtautahi, Waitaha, we consulted with mana whenua prior to commencing the study via the Ngāi Tahu Consultation and Engagement Group for critique and approval of research design and planning, undertook other internal and external Māori consultation, and ensured incorporation of mātauranga Māori and Māori perspectives throughout the study. We also recognised the potential for whakamā when participating in this study due to intergenerational disconnection from language and culture. To mitigate this, we redesigned the TRMDTT user interface to include bilingual instructions and a list of the Māori digits on screen for participants to refer to as needed. We encouraged the attendance and support of whānau members and demonstrated care throughout. In addition to obtaining verbal consent before touching parts of the body (such as the head and ears), time at the beginning and ending of each session was set aside for wairuatanga in the form of karakia.
*7. Kia māhaki (Be humble).* It was important to adopt a learner perspective during recruitment meetings. In all clinical interactions we took care to use clear and straightforward communication, both in the information and consent forms, and in conveying the results of the hearing assessment, adjusting communication styles to the individual (acknowledging their preferences for more or less information).
*8. Kia kaha Te Reo Māori*. TRM was acknowledged as a taonga. We intend that this study and our wider kaupapa contribute to the revitalisation and normalisation of TRM in hearing healthcare. Participant information sheets incorporated TRM throughout the text, and the Māori Speech Phonemes Audiogram of Familiar Sounds was used to help participants understand which speech sounds are inaudible/audible with different hearing abilities.

#### The validation stream

The focus of this stream was the validation of Te Whakamātautau Whakarongo o Aotearoa (Te Reo Māori Digit Triplet Test, or TRMDTT). This stream included population-based health screening principles, clinical tests and procedures, and statistical analyses; important knowledge and techniques derived from Western science that needed to be included in the creation of a hearing screening test. These quantitative methods were used to collect and analyse numerical data to examine the relationships between the test score and the participants’ hearing level, determine the test cut-off level that gives the optimal sensitivity and specificity, and determine the effect of the interaural phase relationship of the stimulus (i.e. diotic or antiphasic) on these measures. However, as mentioned above, the re-emergence of another COVID-19 wave meant that data collection paused to prioritise the health and well-being of communities involved in the study. Data collection in this stream was not resumed until the work of Neame (2024), who followed and built on the template provided by the current project to complete the validation of TRMDTT.

DTTs are increasingly popular worldwide as a speech-in-noise hearing screening method. The tests can be self-administered remotely without specialist equipment, making them cost-effective for hearing screening on a large scale (Smits and Houtgast 2005). The DTT uses three-digit combinations (digit triplets) presented in a steady-state speech noise masker. The rationale for choosing digits as speech stimuli is that they have a high level of intelligibility and familiarity, even for young children and non-native language learners (Ramkissoon et al. 2002).

The TRMDTT uses numbers spoken in te reo which are delivered via headphones to the listener. The speech material features the carrier phrase ‘ko ngā nama’ (‘the numbers’) and the spoken numbers zero to nine. The listener then enters the digits they heard on a keypad. On-screen test instructions were written in te reo alongside the English translation for the listener. We decided to include bilingual instructions and a list of the Māori numbers for participants to refer to as needed to mitigate the risk of participants feeling whakamā if they didn’t understand. We hoped to make the TRMDTT more accessible to all, regardless of their language skill level.

The research design enabled participants to choose the location of their hearing screening. Participants could choose between attending the campus clinic or they could opt for the researcher to visit them in the community, for example, at their local marae (a significant Māori community complex of buildings). This was intended to address some of the systemic and practical barriers to access and participation that disproportionately affect Māori, such as transport issues, getting time off work, financial barriers, and cultural safety issues. Overwhelmingly, participants expressed a preference for community screening rather than attending the university clinic. To overcome the technical challenges of obtaining reliable hearing thresholds in ambient noise, we procured a KUDUwave portable ‘booth-less’ diagnostic audiometer (essentially a computer-based audiometer delivered through highly noise-occluding headphones) for use on community-based projects.

Two-hour hearing screening appointments were scheduled for participants. During these appointments, after appropriate tikanga had commenced, screening procedures were outlined, and questions were encouraged before informed consent was obtained. Firstly, an oral history questionnaire was completed by the participants to help the researcher understand their hearing and balance from their perspective and identify areas of concern. Secondly, verbal consent for head touch was obtained before otoscopy to assess the external auditory canal and tympanic membrane status. The participant chose the test sequence, including pure-tone audiometry, tympanometry, and the TRMDTT.

## Discussion

The COVID-19 pandemic disrupted and altered the course of the original aims of the study. Originally, the study aimed to validate and improve TRMDTT and conduct the study according to Māori cultural values, inspired by He Awa Whiria and the ‘Community-Up’ approach. During the pandemic, recruitment and data collection to validate TRMDTT was stopped, primarily to protect the health of kaumātua, but also because disruptions to global supply chains during the pandemic delayed the arrival of the KUDUwave mobile audiometer, cutting short our opportunity to gather more audiometric data in community settings. This made it difficult to address the study's aims, and as a result the study was statistically underpowered with only 34 participants.

However, this provided an opportunity to critically reflect on approaches and hearing healthcare praxis overall. It was through the approach stream that the integration of cultural safety, tikanga Māori and Māori values within hearing healthcare practice was explored and documented. Examples of praxis are ways in which Tangata Tiriti could adopt Māori protocols and values in their work as clinicians and/or researchers.

This study adds to the emerging research of culturally safe audiological practice. Wepa (2003) summarises cultural safety in nursing as,

the effective nursing of a person/family from another culture by a nurse who has undertaken a process of reflection on [their] own cultural identity and recognises the impact of the nurse’s culture on his or her own nursing practice (340).

Similarly, Brewer et al. (2015) describe three key factors in providing culturally safe speech and language therapy (SLT) including: (1) individual clinician factors, (2) human and non-human resources, (3) practical and cultural ways of working. The present study is the first in the field of hearing health to combine all three factors: the awareness of individual clinician factors, the use of a more culturally appropriate tool and suggested ways of working in audiology research. The evaluation of culturally safe practices is also necessary (Nash et al. 2022).

The development of the TRMDTT and understanding of Māori hearing loss has been an ongoing and incremental process. In reading through written accounts of Māori hearing loss, we were mindful that many authors were non – Māori writing from an ethnographer lens, and as such we considered who the authors were, how they collected data, and why they collected that information. We also noted that the first steps of development of TRMDTT were impeded by a lack of Māori-led, Community-Up development and evaluation. Māori-led kaupapa Māori qualitative studies at the University of Canterbury have been completed to understand participants’ experiences of TRMDTT and hearing healthcare overall (Lowe et al. 2024; Manuel et al. 2024).

There are several strengths to this study. Between COVID-19 lockdowns, we took numerous opportunities to attend community health education events and hui organised by marae and Māori health providers, which are reliant on building and maintaining reciprocal relationships as an ongoing research culture rather than one-off engagement. In some cases, delays meant it was necessary to start from the beginning again, and rebuild relationships via the principle of kanohi kitea (Cram and Pipi 2000; O'Carroll 2013).

One key strength of the TRMDTT is as a ‘low touch’ or ‘no-touch’ method of hearing screening that can be administered remotely (De Sousa et al. 2020). As Exeter (2015) forecasts the prevalence of hearing loss for New Zealanders aged over 65 years will increase more in rural areas, the TRMDTT could provide an additional point of access to screenings in these regions as well as for those who are unable to leave home. Similarly, using the KUDUwave mobile audiometer allows hearing testing to be performed in these remote areas outside the traditional audiological sound booth (Serpanos et al. 2022).

The present study also demonstrated the utility of He Awa Whiria in hearing healthcare. The combined approach successfully identified a broader participant base than in previous studies (Murray 2012; Bowden 2013) and disproved previous assumptions that there were too few te reo speakers in Ōtautahi/Christchurch with hearing loss. Additionally, practical clinician-led behaviour could address some of the often-cited gaps or criticisms of the biomedical model. This resulted in the inclusion of time for whakawhanaungatanga (an indigenous process of creating relational connection); space to perform karakia and time for verbal consent before touching tapu (sacred) parts of the body, such as the head and ears. While this practice was in keeping with various medical professional bodies’ best-practice guidelines on providing healthcare to Māori patients and their whānau, it must be noted that there were no best-practice guidelines specific to the audiology profession. This is a basic standard that requires urgent attention to fulfil the Te Tiriti obligations of the profession.

The braided rivers analogy also highlighted points where the two stream aims began to diverge. The time taken to obtain the audiological and TRMDTT data combined with the time for whakawhanaungatanga and manaakitanga (the process of showing respect, generosity and care for others) led to a long testing session for participants, and our follow-up studies have made adjustments to the processes of both streams to mitigate this (Neame 2024). The He Awa Whiria framework and Kaupapa Māori approaches would be beneficial for hearing healthcare to explore what culturally appropriate hearing assessments might look like, in partnership with Māori. The TRMDTT is a positive first step towards addressing a lack of hearing assessment materials in te reo.

While an obvious next step might be to develop te reo versions of other commonly used hearing screening tools, Māori-led research may prioritise different solutions that are more consistent with an indigenous worldview. For example, kaumātua questioned why spoken digits were chosen as stimulus for the TRMDTT. They suggested nature sounds, such as bird calls or animal sounds, would be more connected to te Ao Māori (the Māori world). Using ecological sound stimuli has been explored by various researchers as an alternative to pure-tones for testing children (Myers et al. 1996; Nolte et al. 2016; Denys et al. 2019); however, the utility of such methods has yet to be explored in the context of culturally appropriate screening materials for indigenous populations. This warrants further investigation using indigenous research methodologies, paradigms, and methods.

## Conclusion

‘*Kua hua te mārama’ [the moon is full]^1^*

Whakataukī/Māori Proverb (In Mead and Grove 2001, p.272).

Māori have been impacted by hearing loss across the life course and across generations, with stories and shifting views of disability. The TRMDTT is a positive first step towards addressing a lack of hearing assessment materials in te reo. This paper demonstrated the utility of He Awa Whiria for the hearing health sector. He Awa Whiria inspired the research team to take an effective Western approach to hearing screening and enrich the research design and data collection processes with Mātauranga Māori and tikanga Māori. Not only did this approach successfully identify a broader participant base than in previous studies, and lead to greater participant engagement (Lowe 2023), it also shows promise as a way for hearing health services to explore what culturally appropriate hearing assessments might look like in partnership with Māori. We suggest that some of the often-cited gaps or criticisms of the biomedical model of hearing assessment could be addressed with the development of best evidence practice guidelines that fulfil Te Tiriti obligations.

## Note

1 This whakatauki was chosen for the conclusion of this study as the saying relates to something that has reached the final stages and is completed.
